# Protocol for the NEURO-BREAC-02 Trial: Evaluation of a Self-Assessment Tool for Mild Chemotherapy-Induced Peripheral Neuropathy in Breast Cancer Survivors

**DOI:** 10.3390/jcm15134881

**Published:** 2026-06-23

**Authors:** Dirk Rades, Maria Karolin Streubel, Christian Staackmann, Laura Doehring, Achim Rody, Maria Joy Normann Haverberg, Martin Ballegaard

**Affiliations:** 1 Department of Radiation Oncology, University of Luebeck, 23562 Luebeck, Germany; 2 Department of Radiation Oncology, University Medical Center Schleswig-Holstein, Luebeck Campus, 23538 Luebeck, Germany; mariakarolin.streubel@uksh.de (M.K.S.); christian.staackmann@uksh.de (C.S.); laura.doehring@uksh.de (L.D.); 3 Department of Obstetrics and Gynecology, University of Luebeck, 23562 Luebeck, Germany; achim.rody@uksh.de; 4 Department of Obstetrics and Gynecology, University Medical Center Schleswig-Holstein, Lübeck Campus, 23538 Lübeck, Germany; 5 Department of Neurology, Zealand University Hospital, 4000 Roskilde, Denmark; mjha@regionsjaelland.dk (M.J.N.H.); mbag@regionsjaelland.dk (M.B.); 6 Department of Clinical Medicine, Faculty of Health and Medical Sciences, University of Copenhagen, 2200 Copenhagen, Denmark

**Keywords:** breast cancer, paclitaxel, docetaxel, chemotherapy-induced peripheral neuropathy, scoring tool

## Abstract

**Background/Objectives:** Patients with breast cancer undergoing chemotherapy with taxanes or platin derivates often develop peripheral neuropathy (CIPN). Higher-grade CIPN generally affects the patient’s quality of life. Because the treatment options for CIPN are limited, early diagnosis is desirable to allow for timely modifications of the treatment. This may be facilitated with scoring tools that are ideally usable by the patients. A prospective study suggested that a recently developed self-assessment tool might be able to reveal the difference between the absence of CIPN and higher-grade CIPN. When CIPN has reached an advanced grade, alteration of the chemotherapy regimen may have only a limited effect. Therefore, it is important to know whether the new scoring tool can identify CIPN even when it is still mild. The current trial (NCT07604441) aims to identify the optimal cutoff point value for detecting mild CIPN. Given the limited sample size, the derived cutoff will be considered preliminary and will require validation in a larger independent cohort. **Methods:** The NEURO-BREAC-02 trial aims to identify the optimal cutoff point value of the new tool to distinguish between absent and mild CIPN after treatment with taxanes for breast cancer. Scores ranging between 0 and 44 points are reported via self-assessment supported by a neuropathy tracker. Secondly, the satisfaction of participants with the self-assessment tool is evaluated. Twenty-six participants (19 with mild CIPN and 7 without CIPN) are required, and 28 must be enrolled. **Conclusions:** The outcomes of the NEURO-BREAC-02 trial are considered crucial for the creation of a self-assessment tool to identify mild CIPN in patients with breast cancer and are a necessary addition to the preceding NEURO-BREAC study.

## 1. Introduction

During the last decades, taxanes such as paclitaxel and docetaxel have increasingly been used for the treatment of early and metastatic breast cancer [1]. In previous trials, the addition of taxanes to anthracycline chemotherapy led to a significant improvement in local control and overall survival [2,3]. Chemotherapy regimens including taxanes frequently lead to peripheral neuropathy (CIPN) [4,5,6,7,8]. Its mechanism is considered to be multi-factorial, preferentially affecting sensory neurons [4,9]. In addition, taxanes can lead to structural alterations in axonal mitochondria and the destruction of microtubules and subsequent impairment of axonal transport and the supply of energy for the cell that may even result in cell death [4,10]. Moreover, reactive oxygen due to mitochondrial dysfunction may result in oxidative stress and consequent neuropathic pain [4,11]. The effect on peripheral nerves may lead to an increased concentration of pro-inflammatory cytokines at the affected sites and the aggravation of allodynia [4].

Moderate-to-severe CIPN is generally associated with neurologic symptoms including numbness, tingling, pain, sensory deficits, balance impairment, and functional limitations. Therefore, this complication can be quite burdensome for the affected patients. In the case of moderate CIPN, symptoms limit the age-appropriate instrumental activities of daily living, e.g., preparing a meal [12]. Severe CIPN is defined as being associated with significant functional impairment, limiting the self-care activities of daily living such as eating and dressing. New hospitalization or the prolongation of existing hospitalization is often required. By contrast, mild CIPN is associated with minor symptoms such as subtle tingling or may even be asymptomatic [12]. According to the Common Terminology Criteria for Adverse Events (CTCAE), an intervention is not indicated for mild CIPN [12].

Because there is currently no effective prophylaxis available to avoid peripheral neuropathy and treatment options are quite limited, it would be desirable to identify CIPN at an early stage, i.e., when it is still mild [8,13,14,15]. This would allow for adjustment of the treatment regimen and reduce the probability of progression to moderate and severe CIPN [14,15,16,17,18,19]. Scoring tools would be helpful in that regard. Commonly used tools require the presence and action of specifically trained health care professionals plus special neurological equipment [20,21,22,23,24,25,26,27,28]. A tool usable by the patients alone, i.e., not requiring support from health care professionals, would be more convenient. Recently, such a scoring instrument considering patient-reported neurologic symptoms and outcomes of self-assessment procedures performed by the patients has been created and evaluated in a series of patients with peripheral polyneuropathy of any cause [29,30].

Moreover, we have performed a prospective trial in patients with higher-grade (moderate-to-severe) CIPN after systemic treatment with taxane-based chemotherapy for breast cancer [31,32]. In that trial, we were able to define the optimal cutoff point value to delineate higher-grade CIPN from the absence of CIPN. Both the external reviewers and members of our study group asked the same question: would the new scoring tool also be able to distinguish between absent and mild CIPN? Therefore, the present NEURO-BREAC-02 trial was created. Its major goal is to find the cutoff point value of the new tool with the highest accuracy to identify patients with breast cancer experiencing mild CIPN. The diagnosis of CIPN at an earlier stage would ideally enable the treating physicians to adjust the prescribed chemotherapy regimen in a timely manner to avoid higher-grade CIPN. The protocol of the NEURO-BREAC-02 trial was approved by the competent ethics committee on 13 May 2026 (file 2026-138) and registered at clinicaltrials.gov on 21 May 2026 (identifier NCT07604441). The outcomes of the NEURO-BREAC-02 trial are expected to be a valuable contribution to the development of a self-assessment tool that will help diagnose mild CIPN in patients with breast cancer during or following treatment. This study is a necessary addition to the preceding NEURO-BREAC study to achieve this goal.

## 2. Design and Materials

### 2.1. Objectives

The main goal (primary endpoint) of this trial is to identify the most appropriate cutoff point value of a new tool to discriminate between mild and absence of CIPN after taxane-based chemotherapy and adjuvant radiotherapy for breast cancer. Mild and absent CIPN were identified with the clinical Total Neuropathy Score (TNS) as described in Section 2.5.4 [15,16,17,18,19,20,21,22,23,24,25,26,27,28]. The point values of the new tool (0 to 44 points) are received with the help of a neuropathy tracker [29,30]. The receiver operating characteristic (ROC) curve is used to show the connection between sensitivity and specificity for every possible cutoff point value and to select the optimal value for detecting mild CIPN. Secondary endpoints are satisfaction with the scoring system and identification of an optimal cutoff point value for the Utah Early Neuropathy Scale (UENS) to discriminate between mild CIPN and no CIPN [20].

### 2.2. Trial Design and Duration

This is a mono-center single-arm prospective trial. Recruitment of all 28 participants should be completed within 1.5 months. Because time until completion of the trial procedures will be up to two weeks, total running time will be about two months.

### 2.3. Patient Selection

Participants must be informed about nature, significance and scope of the trial. They may only be included if they meet all inclusion criteria and do not meet any of the listed exclusion criteria (Table 1).

### 2.4. Stopping Rules

The study participant may withdraw her consent any time and without stating a reason. The trial ends after completion of study procedures by the last participating patient. The principal investigator may stop the trial prematurely due to safety reasons. In this case, patients and responsible ethics committee are informed immediately.

### 2.5. Treatment and Trial Procedures

Initially, medical history, co-morbidities, current medication, demographics, body mass index, performance status, tumor stage, tumor type, and treatment-related aspects will be recorded (Table 2).

#### 2.5.1. Chemotherapy Regimens

Prior to this study, the patients had received chemotherapy including paclitaxel or docetaxel. The chemotherapy regimens qualifying for this trial have been reported before [31,32,33].

#### 2.5.2. Self-Assessment of CIPN by the Patients

The patients rate their symptoms and signs of neuropathy with the support of the neuropathy tracker (Table 2) [20]. They receive a mobile phone with the corresponding software to go through the self-assessment procedure themselves. After training and instructions from a member of the trial team, the patients perform the self-assessment in the presence of team members to minimize the risk of errors [29,30]. Initially, the tracker asks the user about quality and intensity of symptoms in the legs likely related to CIPN (0 to 4 points in total). Subsequently, the tracker guides the user through a systematic procedure of pinprick using a safety pin in six skin areas of each leg, starting at the toes and ending at the knee (0 to 24 points in total). In the allodynia/hyperalgesia section, painful sensation to prick or touch is assessed (0 to 4 points in total). Moreover, vibration is assessed with a mobile phone at three predefined points of each leg (0 to 6 points in total), as well as feeling of modifications of the joint of the big toes (0 to 2 points in total). In the last section, the patients have to assess the strength of their big toes as represented by their extension (total of 0 to 4 points). In general, the procedure of self-assessment considers many aspects of the UENS [20]. The self-assessment by the patients is performed prior to the examinations by members of the study team (TNS, UENS) to avoid a corresponding bias during the self-assessment procedure. Blinding of the trial team members who apply the TNS and the UENS is not planned.

#### 2.5.3. Satisfaction with the Scoring System

Patients complete a questionnaire regarding their satisfaction with the scoring tool (Table 2) [34,35]. If >20% of the patients are not satisfied, the tool must be optimized prior to further use. If >40% are not satisfied, the tool is rated as not usable to help identify mild CIPN in breast cancer survivors. The questionnaire includes five questions. The patients are asked to state whether they consider the tool comprehensible, easy to handle, helpful, clear, and associated with a feeling of safety [34,35]. For each item, the patients can assign 1 to 7 points (7 points = most positive answer). In accordance with previous studies, satisfaction is defined as a mean score of at least 4.0 points [32,36].

#### 2.5.4. Assessment of CIPN with TNS and UENS by Health Care Professionals

The clinical TNS is used for the screening procedure, namely, to identify patients with absent or mild CIPN. It considers severity of sensory and motor symptoms, autonomic deficits, decreased pin and vibration sensitivity, motor deficits, and decreased reflexes [25,26,27,28]. Depending on severity, 0 to 4 points are given for each symptom, which results in total scores of up to 28 points. Absence of peripheral neuropathy is represented by a total score of 0–1 points, considering that the parameter “autonomic symptoms” includes symptoms likely not related to CIPN, and mild peripheral neuropathy is represented by 2–7 points [28].

For the UENS, a specific safety pin and a tuning fork are needed [20]. The UENS covers the motor examination, pin sensation, allodynia/hyperesthesia, large fiber sensation, and deep tendon reflexes sections for both legs. Maximum achievable points for these sections are 4, 24, 2, 8, and 4 points, respectively, resulting in a maximum total score of 42 points.

#### 2.5.5. Safety Management

Adverse events are not expected from participation in the NEURO-BREAC-02 trial, because the study procedures last only about one hour and do not include interventions that have to be considered potentially harmful. An adverse event is an event that is not necessarily treatment-related. Events covered by the documentation for co-morbidities, CIPN, or treatment-associated toxicity Grade ≤ 2 do not require additional documentation. If concomitant diseases appear after the start of the study, an adverse event form has to be completed. Unexpected adverse events are those whose type, frequency and degree of severity are not expected based on current knowledge. Serious adverse events are life-threatening or lethal, require admittance to hospital, lead to significant disability, or are considered medically relevant. Serious adverse events and unexpected adverse events must be reported within 24 h to the principal investigator. For assessment of the severity of adverse events, the Common Terminology Criteria for Adverse Events version 5.0 is used [12].

### 2.6. Statistics

#### 2.6.1. Calculation of the Sample Size

The discriminative power of the scoring tool is determined by calculating the area under the curve (AUC) of the receiver operating characteristic (ROC) curve. The sample size should allow for acceptable precision in the estimation of the AUC measured by means of the 90% confidence interval by incorporating a pre-defined probability [37]. Calculation of the sample size is based on a two-sided level of significance of 10%, an AUC of 0.95, a lower bound of the confidence interval of 0.7, assurance probability of 80%, and a ratio of participants with absence of CIPN to participants with mild CIPN of 0.33. The assumed AUC of 0.95 represents a planning assumption reflecting the level of diagnostic discrimination considered clinically meaningful for further development of the scoring tool rather than a definitive expectation of the final performance. This assumption is supported by the preceding NEURO-BREAC trial, which suggested promising discriminative properties of the tool for identifying moderate-to-severe CIPN. Nevertheless, distinguishing mild CIPN from absence of CIPN is clinically more challenging; therefore, the present study is considered exploratory and is intended to generate a preliminary cutoff to be validated in subsequent larger studies.

Based on these assumptions above, 26 patients (19 with mild CIPN and 7 without CIPN) are needed for the full analysis set. Assuming that roughly 5% will not be suitable for this set, 28 patients need to be recruited for the NEURO-BREAC-02 trial.

#### 2.6.2. General Statistical Calculations

Baseline characteristics, rates of mild CIPN, and safety are analyzed descriptively. Categorical data are reported in contingency tables, and continuous data are summarized considering frequency, mean (plus standard deviation), and median (plus quartiles and range). Protocol deviations are considered and reported. Data analyses follow an a priori statistical analysis plan. Statistical analyses will be performed with version 9.4 of SAS software (SAS Institute Inc., Cary, NC, USA).

#### 2.6.3. Statistical Considerations Regarding the Primary Endpoint

The primary aim is to identify the optimal cutoff point value of a scoring tool to distinguish between mild and absent CIPN. Initially, sensitivity and specificity are provided for every possible cutoff point value. The ROC curve is used to illustrate the relation between sensitivity and specificity. A ROC curve demonstrating a greater discriminant capacity of the scoring tool is located closer to the upper-left-hand corner. The AUC summarizes the entire location of the ROC curve. In case of an AUC of 1.0, the cutoff point value is perfect with respect to demarcation between mild and absent CIPN. Given the small number of participants expected in the no-CIPN reference group, the ROC analysis will be interpreted with caution.

If statistical significance of the AUC is reached, the most appropriate cutoff point value with a sensitivity ≥90% and a specificity ≥80% is going to be established. Moreover, the Youden index (sensitivity + specificity − 1) will be calculated. If the observed AUC is lower than anticipated, particularly below the pre-specified threshold of 0.70 for the lower confidence bound, no definitive diagnostic cutoff will be claimed; instead, results will be used to inform refinement of the scoring tool and planning of a larger validation study.

In addition, the relation between tertiles of the cutoff point values and mild CIPN will be evaluated with the Jonckheere–Terpstra test, which investigates the global null hypothesis that the distribution of the variable is not different between the tertiles. It is designed to identify alternatives of ordered differences, i.e., that the occurrence of mild CIPN increases with the tertiles of cutoff point values.

For the primary analysis, the full analysis set will include all participants with a complete neuropathy tracker assessment and a conclusive TNS-based classification as either no CIPN or mild CIPN. Because the primary endpoint is the identification of a diagnostic cutoff, missing items of the neuropathy tracker will not be imputed. If the total tracker score cannot be calculated reliably, the participant will be excluded from the primary endpoint analysis. TNS serves as the reference assessment for the classification of CIPN status. Participants with incomplete TNS assessments that do not allow reliable classification as no CIPN or mild CIPN will not be included in the primary ROC analysis.

#### 2.6.4. Statistical Considerations Regarding the Secondary Endpoints

Satisfaction with the scoring tool is evaluated following the completion of the self-assessment of symptoms and signs of neuropathy using the corresponding tracker (Table 2). If >20% of the participants are not satisfied, the tool must be optimized prior to further use. If >40% are not satisfied, the tool is rated as not usable [31]. Satisfaction with the scoring tool will be analyzed descriptively. The total satisfaction score will be calculated only for participants with complete responses to all satisfaction items. The number and proportion of missing or incomplete assessments will be reported for each endpoint.

The statistical methods applied to the primary endpoint will also be performed with respect to the UENS. Spearman rank correlation will be the primary correlation measure. Pearson correlation will be reported as an exploratory measure of linear association only if visual and descriptive assessments support approximate linearity and normality. Linearity will be assessed by scatterplots, and distributional assumptions will be explored using histograms, Q-Q plots, and the Shapiro–Wilk test.

Because correlation does not assess agreement between instruments, Bland–Altman analyses will be performed descriptively to visualize systematic differences between the neuropathy tracker and UENS scores across the measurement range. For each patient, the difference between the two scores and their average will be computed. The Bland–Altman plot will display differences versus averages and include lines for the mean difference (bias) and limits of agreement, which indicate the range within which most individual differences are expected to lie under approximate normality. The 95% confidence interval for the mean difference will enable the distinguishing of systematic bias (shift) from random variation. Because the neuropathy tracker and UENS are related but distinct instrument tools with slightly difference scoring ranges and assessment conditions, these analyses will not be interpreted as evidence of interchangeability but rather as an exploratory description of bias, variability, and possible score-dependent difference. Moreover, the potential effect of other factors such as concomitant diseases that may lead to peripheral neuropathy, beta blocker treatment, age, gender, body mass index, performance status, and details of the administered chemotherapy (timing: neoadjuvant vs. adjuvant, number of cycles, duration, and cumulative taxane dose) will be exploratorily assessed using Chi-square tests or Fisher’s exact tests. Secondary analyses will be performed using complete paired observations only. Missing UENS items will not be imputed.

### 2.7. Ethical and Legal Principles

The study protocol, the patient information document, and the informed consent form have been approved by the competent ethics committee. This study is conducted in accordance with the Declaration of Helsinki. Prior to inclusion in the trial, patients are fully informed about the trial. After the study participant has given written consent, she receives a copy of both the patient information and the signed informed consent form. Participants are informed that collected data will be documented anonymously and will then be forwarded for scientific evaluation. This is also the appropriate time to indicate the patient’s consent to data protection.

### 2.8. Data Handling and Protection

Data are collected in accordance with the Data Protection Act. The data obtained with a neuropathy tracker are stored on a smartphone, which is provided by the participating trial center and kept under lock and key in this department until the trial is terminated. The data regarding screening, baseline assessment, and findings from the trial are stored on the server of the University Medical Center Schleswig-Holstein at the Lübeck Campus and treated with utmost confidentiality. Access to data is limited to authorized persons including principal investigator, external monitors, and trained staff members actively involved in the study, who have signed a confidentiality agreement and a data protection declaration. In addition, an anonymized Excel spreadsheet needs to be forwarded to the involved external statistician for the analyses of the data according to the pre-defined statistical analysis plan. Data of study participants are recorded in a pseudonymous way. An identification list of study participants is kept solely in the participating institution. For collection of the data, the corresponding documentation sheets are used.

The data are handled in accordance with the General Data Protection Regulation. The originals of all key trial documents are kept at the trial headquarters, i.e., at the institution of the sponsor, for a minimum of 10 years after the final report. The principal investigator keeps all administrative documents (written correspondence with the ethics committee, regulatory authorities, trial management, trial headquarters), the patient identification list, the signed informed consent forms, copies of the documentation sheets, and general trial documents (protocol, amendments). Original patient data (patient files) must also be kept for the length of time stipulated for the trial centers but not for less than 10 years.

Clinical on-site monitoring is conducted in accordance with Good Clinical Practice regulations and standard operating procedures. The site is visited by a monitor, depending on the status of recruitment and data quality. Informed consent and defined key data are checked for all patients. Patient questionnaires are checked for their existence. Regular audits and inspections by federal authorities are not planned.

### 2.9. Dissemination of Results and Publication Policy

The main principal investigator, the external statistician, and staff members involved in the trial will create a study report to be submitted to the responsible ethics committee within twelve months following the official termination of the trial. The findings of the trial are planned to be published in a peer-reviewed journal. In the corresponding publications, the acronym NEURO-BREAC-02 will be mentioned.

## 3. Expected Results Plus Discussion

CIPN is a frequent complication of taxane- and/or platin-based chemotherapy for breast cancer [4,8,19,38]. Moderate and severe CIPN are generally associated with significant neurologic symptoms that affect the patient’s quality of life [39,40,41,42,43]. Unfortunately, the treatment options are very limited. According to a guideline of the American Society of Clinical Oncology, duloxetine, an inhibitor of serotonin uptake and nor-epinephrine, was the only agent with some evidence to be used to treat CIPN [14]. In a randomized trial, it led to an improvement of symptoms of CIPN, including pain, numbness, and tingling of the feet [15]. Given the limited treatment options for CIPN, early diagnosis of this complication is desirable [14,15,16,17,18,19]. Achievement of this goal will likely be facilitated with corresponding scoring tools. Common tools like the UENS and TNS need to be applied by health care professionals, which limits their usability [20,21,22,23,24,25,26,27,28]. A scoring tool applied by the patients themselves appears to be a better option. A self-assessment tool has already been created [29,30]. Moreover, a prospective pilot study showed promising results and suggested that the new tool is able to distinguish between the absence of CIPN and higher-grade CIPN [32]. However, as stated above, the identification of CIPN at a more advanced stage may be too late to alter the currently administered chemotherapy in order to avoid further progression of CIPN. It would be interesting to know whether the new scoring tool is also able to distinguish between absent and mild CIPN.

Therefore, the present NEURO-BREAC-02 trial was developed. Its main goal is to find the optimal cutoff point value for detecting mild CIPN. We expect that this goal will be achieved, which will be important for designing a subsequent prospective trial further investigating the value of the new tool for the early detection of CIPN in patients with breast cancer during a chemotherapy course, immediately after chemotherapy, or during the period of follow-up.

However, when developing the protocol of the subsequent trial, the limitations of the NEURO-BREAC-02 study should be noted. It has been designed to receive initial evidence regarding the ability of the new tool to identify patients experiencing mild CIPN. Because all patients included in the NEURO-BREAC-02 trial will be treated with taxane-based chemotherapy for breast cancer, the new tool may not be usable in patients receiving a different treatment regimen, e.g., including cisplatin or oxaliplatin [38]. The same applies to male breast cancer patients and patients receiving chemotherapy for malignancies other than breast cancer. Additional limitations include the mono-centric nature of the trial and its comparably small sample size. Another limitation is the small number of participants expected to have no CIPN, which may affect the precision of the specificity estimates and the stability of the ROC curve. Thus, the derived cutoff should be considered exploratory and will require confirmation in a larger independent validation cohort. In addition, the assumed AUC used for sample size planning reflects a target level of performance considered clinically relevant for further development of the tool. If the observed diagnostic performance is lower, the results will still provide important information for refining the scoring system and designing subsequent validation studies. Moreover, due to the low number of patients planned to be recruited, stratified analyses by baseline factors and subgroup analyses appear not to be justified. The statistical power to identify heterogeneity would not be sufficient. Moreover, the informative value of the assessment of the influence of patient- and disease-related characteristics may be limited due to the small sample size.

The NEURO-BRAC-02 trial aims to provide initial prospective evidence regarding the value of the new scoring tool. Its major goal is the identification of the optimal cutoff point value to differentiate between absent and mild CIPN. Its findings will be very important and a necessary addition to the preceding NEURO-BREAC study for the creation of a self-assessment tool to identify mild CIPN in patients with breast cancer. If the new tool proves to be sufficiently precise with respect to identifying CIPN at an early stage, i.e., mild CIPN, it will be a valuable diagnostic instrument. It could help guide the modification of chemotherapy doses, referrals to a neurologist, or supportive care interventions. When available, the new tool may be easily implemented into clinical routine, because it can be used by the patients themselves, without specific equipment. It requires only a mobile phone for the assessment of vibration sensitivity. The NEURO-BREAC-02 trial represents an important step for the development of the new tool.

## Figures and Tables

**Table 1:** Inclusion and exclusion criteria.

**Inclusion criteria** Histologically proven breast cancer. Status after irradiation preceded by chemotherapy including taxanes. Mild or absence of peripheral neuropathy according to TNS. Female gender. Aged at least 18 years. Written informed consent. Ability to consent.
**Exclusion criteria** Significant dermatologic disorders. Pregnancy, lactation. Supposed non-compliance.

**Table 2:** Assessments for screening at baseline and at the day the study procedures are performed.

	Screening	Baseline	Day of Study Procedures
Demographics	X		
Medical history and concomitant diseases		X	X
Concomitant medication		X	X
Chemotherapy details	X		
Radiotherapy	X		
Physical examination		X	X
Body mass index		X	
Performance status		X	
Pathology and tumor stage		X	
Upfront surgery		X	
Informed consent	X		
Inclusion criteria	X		
Exclusion criteria	X		
Scoring tool (by study participants)			X
Satisfaction with the tool			X
UENS (by health care professionals)			X
TNS (by health care professionals)			X

## Data Availability

Additional information is available at clinicaltrials.gov (NCT07604441, registered on 21 May 2026).

## Abbreviations

AUC	Area under the curve
CIPN	Chemotherapy-induced peripheral neuropathy
ROC	Receiver operating characteristic
TNS	Total neuropathy score
UENS	Utah Early Neuropathy Scale
